# Does Basis Set Superposition Error Significantly Affect Post‐CCSD(*T*) Corrections?

**DOI:** 10.1002/jcc.70007

**Published:** 2024-12-24

**Authors:** Vladimir Fishman, Emmanouil Semidalas, Margarita Shepelenko, Jan M. L. Martin

**Affiliations:** ^1^ Department of Molecular Chemistry and Materials Science Weizmann Institute of Science Rehovot Israel

**Keywords:** basis set superposition error (BSSE), noncovalent interactions, post‐CCSD(*T*) correlation effects, thermochemistry

## Abstract

We have investigated the title question for both a subset of the W4‐11 total atomization energies benchmark, and for the A24x8 noncovalent interactions benchmark. Overall, counterpoise corrections to post‐CCSD(*T*) contributions are about two orders of magnitude less important than those to the CCSD(*T*) interaction energy. Counterpoise corrections for connected quadruple substitutions (*Q*) are negligible, and ${\left(Q\right)}_{\Lambda }-\left(Q\right)$ or $T_{4}-\left(Q\right)$ especially so. In contrast, for atomization energies, the $T_{3}-\left(T\right)$ counterpoise correction can reach about 0.05 kcal/mol for small basis sets like cc‐pVDZ, thought it rapidly tapers off with cc‐pVTZ and especially aug‐cc‐pVTZ basis sets. It is reduced to insignificance by the extrapolation of $T_{3}-\left(T\right)$ applied in both W4 and HEAT thermochemistry protocols. In noncovalent dimers, the differential BSSE on post‐CCSD(*T*) correlation contributions is negligible even in basis sets as small as the unpolarized split‐valence cc‐pVDZ(no d).

## Graphical abstract

As can be seen in the above box plot, the counterpoise correction on the post‐CCSD(T) corrections is over an order of magnitude smaller than that on the connected triples, and two orders smaller than that on the CCSD correlation energy.

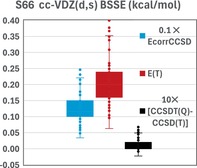

## 1 Introduction

Basis set superposition error (BSSE) is the error in the interaction energy between, for example, a dimer *AB* and its constituent monomers *A* and *B* when evaluated in a finite basis set. (At the complete basis set (CBS) limit, BSSE vanishes.) The classic remedy for BSSE is the Boys–Bernardi counterpoise (CP) method [1].
(1)

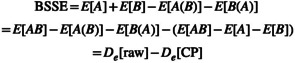

where $D_{e}$ denotes the dissociation energy, $E\left[\mathrm{AB}\right]$ is the total energy of the dimer, $E\left[A\left(B\right)\right]$ the total energy of *A* in the presence of the basis functions on *B*, $E\left[A\right]$ the corresponding total energy in their *absence*, and so forth.

Inclusion of BSSE in noncovalent interaction (NCI) studies is more or less standard operating procedure, especially in smaller and medium basis sets, as the CP corrections may be on the same order or magnitude as the interaction energies of interest.

**FIGURE 1:**
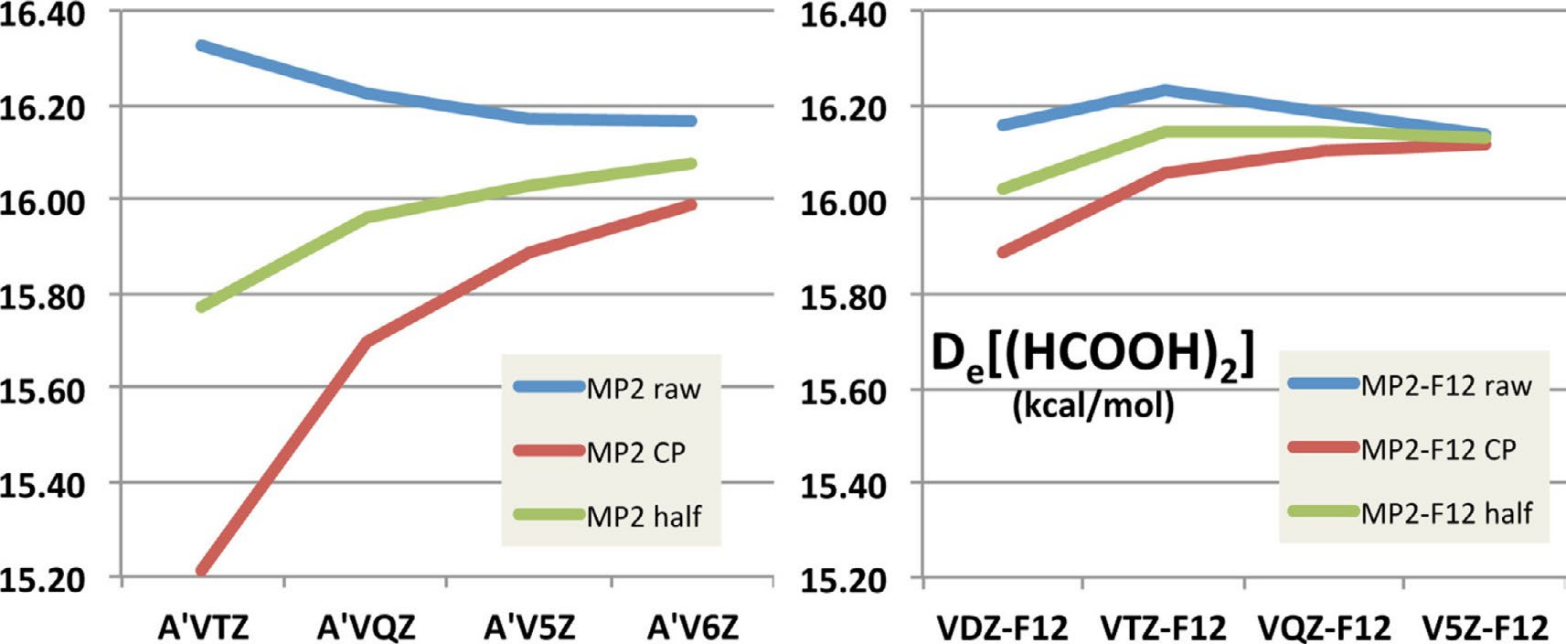
Illustration of effect of BSSE on basis set convergence for formic acid dimer. Reprinted from figure 1 in Reference [2], with Creative Commons license.

Now it is indeed true that full counterpoise does *not* guarantee hewing closer to the CBS limit: as shown by Burns, Marshall, and Sherrill [3] for orbital WFT calculations, and by Brauer, Kesharwani, and Martin [2] for explicitly correlated [4, 5, 6] F12 calculations, error compensation may take place between BSSE (which always overbinds) and IBSI (intrinsic basis set incompleteness, which almost invariably underbinds). Hence, for small basis sets, complete neglect of BSSE may actually be beneficial, and for medium‐size basis sets, “half‐counterpoise” (average of corrected and uncorrected interaction energies) tends to offer superior performance [2, 3, 7]. See Figure 1 for an illustration.

In computational thermochemistry, however, the IBSI overwhelms BSSE to such an extent that most researchers make no effort to apply BSSE corrections. This is particularly the case for total atomization energies (TAE), which are the quantum chemical “cognates” of heats of formation $\Delta H_{f}^{\circ }$. The “raw” and CP‐corrected TAEs are defined analogously to the NCI situation as (e.g., for a triatomic):

(2) $$\begin{array}{c} TAE_{\mathrm{raw}}\left[\mathrm{ABC}\right]=E\left[A\right]+E\left[B\right]+E\left[C\right]-E\left[\mathrm{ABC}\right]\\ TAE_{\mathrm{CP}}\left[\mathrm{ABC}\right]=E\left[A\left(\mathrm{BC}\right)\right]+E\left[\left(A\right)B\left(C\right)\right]+E\left[\left(\mathrm{AB}\right)C\right]-E\left[\mathrm{ABC}\right]\\ \Delta_{\mathrm{CP}}TAE_{\mathrm{CP}}\left[\mathrm{ABC}\right]=TAE_{\mathrm{raw}}\left[\mathrm{ABC}\right]-TAE_{\mathrm{CP}}\left[\mathrm{ABC}\right]\\ =E\left[A\right]+E\left[B\right]+E\left[C\right]-E\left[A\left(\mathrm{BC}\right)\right]-E\left[\left(A\right)B\left(C\right)\right]-E\left[\left(\mathrm{AB}\right)C\right] \end{array}$$

where the Wells and Wilson [8] SSFC (site‐site function counterpoise) *n*‐body generalization of Equation (1) has been applied. (This effectively amounts to evaluating each atomic energy in the full molecular basis set.)

Higher‐accuracy computational thermochemistry protocols like ccCA [9, 10] by the Wilson group, HEAT by the Stanton group [11, 12, 13, 14], Weizmann‐*n* by our own group [15, 16, 17], and FPD (Feller–Peterson–Dixon, see References [18, 19] and references therein) all entail some variant of basis set extrapolation. If the latter works properly, it ought to eliminate BSSE altogether. (Indeed, we recently [20] exploited this fact to “reverse‐engineer” basis set extrapolations.)

Studies of noncovalent interactions, with rare exceptions (such as References [21, 22, 23]) stick to the CCSD(*T*) [24, 25] “gold standard of quantum chemistry” and ignore post‐CCSD(*T*) corrections. However, in thermochemistry, especially for TAEs, it is well‐known (see References [11, 26] for early reports) that kJ/mol accuracy cannot be achieved without them. CCSD(*T*) in fact outperforms the more rigorous CCSDT owing to a well‐established error compensation (e.g., References [11, 13, 15, 26]): higher‐order triples, $T_{3}-\left(T\right)$, are almost always antibonding, while connected quadruples (*Q*) are universally bonding.

On the one hand, CCSDT(*Q*) and especially CCSDTQ have very steep CPU time scalings of $O\left(n_{\mathrm{occ}.}^{4}N_{\text{virt}.}^{5}\right)$ and $O\left(n_{\mathrm{occ}.}^{4}N_{\text{virt}.}^{6}\right)$, respectively. On the other hand, these higher‐order corrections converge much more rapidly with the basis set than the overall correlation energy [16]. In response to a reviewer comment, we offer Table 1 as an illustration, compiled from data in the supporting information of Reference [28]. The statistics given there cover a 65‐molecule subset of the 200‐molecule W4‐17 thermochemical benchmark [27]; the subset spans a broad range of nondynamical (static) correlation character, from essentially pure dynamical correlation in H_2_O and CH_4_ at one end, to strong static correlation in O_3_, singlet C_2_, and BN at the other end. It is clearly seen in Table 1 that RMS contributions taper off rapidly as the connected excitation level increases, to reach insignificance beyond CCSDTQ(5)_Λ_. In tandem, it is also seen that basis set convergence becomes ever more rapid, with even unpolarized cc‐pVDZ(p, s) yielding surprisingly small errors beyond CCSDT(*Q*), and ultimately dwindling down into numerical noise.

**TABLE 1:** Basis set convergence of RMS post‐CCSD(*T*) contributions to the TAEs (kcal/mol) of the 65‐molecule W4.3 subset of the W4‐17 thermochemical benchmark [27].

	$T_{3}-\left(T\right)$	$\left(Q\right)$	$T_{4}-\left(Q\right)$	${\left(Q\right)}_{\Lambda }-\left(Q\right)$	$T_{4}-{\left(Q\right)}_{\Lambda }$	${\left(5\right)}_{\Lambda }$	$T_{5}-{\left(5\right)}_{\Lambda }$	${\left(6\right)}_{\Lambda }$	$T_{6}-{\left(6\right)}_{\Lambda }$	$T_{7}$
nihil	0.84	1.29	0.26	0.17	0.11	0.08	0.015	0.009	0.001	0.001
cc‐pVDZ(p, s)	0.53	0.41	0.09	0.07	0.06	0.02	0.003	0.002	0.001	REF
cc‐pVDZ(d, s)	0.54	0.25	0.03	0.03	0.01	0.01	REF	REF	REF	
cc‐pVTZ(f, p)	0.18	0.11	0.012	0.006	0.007	REF	—	—		
cc‐pVQZ(g, d)	0.09	0.04	REF	REF	REF	—	—	—		
cc‐pV5Z(h, f)	0.05	0.02								
cc‐pV{Q, 5} Z	REF	REF								

*Note:* The underlying data were extracted from the ESI of Reference [28], except for the connected sextuples with the cc‐pVDZ(d, s) basis set and septuples with the cc‐pVDZ(p, s) basis set, which were calculated for the present paper using the general coupled cluster implementation [29, 30] in MRCC [31]; raw energies can be found in the present paper's ESI.

Consequently, post‐CCSD(*T*) corrections tend to be evaluated in very small basis sets, and subsequently applied additively. For instance, in W4 theory [15], the $T_{3}-\left(T\right)$ correction is extrapolated from cc‐pVDZ and cc‐pVTZ basis sets (commonly indicated by the shorthand cc‐pV{D, T}Z), the (*Q*) term is evaluated in a cc‐pVTZ basis set, and $T_{4}-\left(Q\right)$ in just a cc‐pVDZ basis set. (In W4lite theory, just CCSDT(*Q*)/cc‐pVDZ is done for the quadruples.)

This then leads us to the main research question of the present paper: are such corrections materially affected by BSSE corrections? The issue was raised by a reviewer of Reference [23], where we showed that post‐CCSD(*T*) contributions for cohesive energies of water clusters approach 1 kcal/mol for isomers of (H_2_O)_20_.

## 2 Computational Details

The CCSDT(*Q*) [32], CCSDT [33], CCSD(*T*)_Λ_ [34, 35, 36, 37], and CCSD(*T*) calculations reported in this work were carried out using a combination of the MOLPRO 2024.1 [38], CFOUR [39], and MRCC [31] electronic structure program systems, run on the CHEMFARM cluster of the Faculty of Chemistry at Weizmann. Owing to issues with inconsistent UHF solutions in the presence of ghost atoms, many of the small‐molecule counterpoise data were generated using the MRCC interfaces of MOLPRO or CFOUR. For the noncovalent interactions, only closed‐shell species are involved, and hence these calculations were carried out using standalone CFOUR (as memory permitted) or MRCC. The latter code was likewise used for some of the additional data in Table 1, using the algorithms presented in References [29, 30].

Reference geometries for the W4‐11 thermochemical benchmark [40] (which is a subset of the larger and more recent W4‐17 database [27]) were taken from the ESI of the W4‐17 paper and used “as is.” Reference geometries for the A24 [21] and S66 [41] datasets were downloaded from the BEGDB database [42] of noncovalent interaction geometries. Using a Python program written by one of us (ES), geometries for A24x8 were generated by compressing or stretching the intermonomer distances by the eight factors {0.9, 0.95, 1.0, 1.05, 1.1, 1.25, 1.50, 2.0} from the familiar S66x8 database [41]. They are provided in the Supporting Information.

For dissociation energies, $D_{e}$, of diatomic molecules, we applied the standard Boys–Bernardi counterpoise definition. For the TAE_*e*_ values (total atomization energies) of small polyatomics, we used the SSFC (site‐site function counterpoise) generalization of Wells and Wilson [8].

The basis sets used are of the correlation consistent [43] family, ranging from cc‐pV*n*Z (*n* = D, T, Q, 5) [44, 45] to aug‐cc‐pV*n*Z (*n* = D, T, Q, 5) [46]. The shorthand cc‐pVDZ(d, s) refers to at most d and s functions, respectively, on nonhydrogen and hydrogen atoms (the full cc‐pVDZ basis set would correspond to cc‐pVDZ(d, p)).

## 3 Results and Discussion

### 3.1 Total Atomization Energies

#### 3.1.1 Initial Check for Diatomic Molecules

RMS (root mean square) BSSE corrections for a sample of 24 heavy‐atom diatomics and 10 diatomic hydrides are given in Table 2.

**TABLE 2:** RMS BSSE corrections (kcal/mol) to post‐CCSD(*T*) $D_{e}$ contributions for a set of 24 AB and 10 AH diatomics.

	(*T*)	${\left(T\right)}_{\Lambda }-\left(T\right)$	$T_{3}-\left(T\right)$	$T_{3}$	$\left(Q\right)$	*T*(*Q*) − (*T*)	${\left(Q\right)}_{\Lambda }-\left(Q\right)$
cc‐pVDZ	0.286	0.008	0.043	0.329	0.004	0.045	0.000
cc‐pVTZ	0.170	0.005	0.013	0.183	0.006	0.019	0.000
cc‐pV{D, T}Z^a^			0.008		0.008		
cc‐pVQZ	0.071	0.002	0.006	0.067	0.003	0.004	0.000
cc‐pV5Z	0.034	0.001	0.006	0.028	0.002	0.004	0.000
haVDZ	0.214	0.008	0.026	0.238	0.002	0.026	0.000
haVTZ	0.077	0.003	0.008	0.070	0.004	0.006	0.000
haVQZ	0.037	0.001	0.010	0.028	0.001	0.009	0.000

^a^ Extrapolation exponents from table 5 of Reference [47].

First of all, unsurprisingly, the effect of BSSE on the difference between CCSDT(*Q*)_Λ_ and CCSDT(*Q*) is less than 0.001 kcal/mol, and can be entirely neglected. For the difference between CCSD(*T*)_Λ_ and CCSD(*T*) we find 0.008 kcal/mol for the cc‐pVDZ and haVDZ basis sets, which tapers down to 0.001 kcal/mol for haVQZ and cc‐pV5Z.

For connected quadruples (*Q*), the RMS BSSE is less than 0.01 kcal/mol RMS even with the cc‐pVDZ basis set, and smaller still for cc‐pVQZ (0.003) and haVQZ (0.001 kcal/mol). We can hence conclude that BSSE on connected quadruples is negligible even for the purposes of high‐accuracy work.

The situation for higher‐order connected triples $T_{3}-\left(T\right)$, however, is somewhat different. For the cc‐pVDZ basis set, we find 0.043 kcal/mol (i.e., 0.18 kJ/mol), which however drops to 0.026 kcal/mol when diffuse functions are added, and to 0.013 kcal/mol when we move things one notch up to cc‐pVTZ. Both W4 and HEAT apply cc‐pV{D, T}Z extrapolations to the higher‐order triples. If we do so here (with extrapolation parameters taken from table V in Reference [47]), the RMS BSSE is just 0.008 kcal/mol, which may be justifiable to neglect in view of other, larger sources of uncertainty such as residual basis set incompleteness in the CCSD(*T*) component [17]. In fact, for the cc‐pV{T, Q}Z basis sets used in W4.3 theory, the BSSE will be even more negligible.

Upon comparing RMS BSSE corrections for (*T*) and for all of $T_{3}$ (i.e., the difference between CCSDT and CCSD), we note that for smaller basis sets like cc‐pVDZ, cc‐pVTZ, and haVDZ, there is more BSSE on $T_{3}$ than on (*T*). For larger basis sets, however, the roles are reversed.

In addition, if one considers the whole CCSDT(*Q*) − CCSD(*T*) difference, one finds partial mutual cancelation for BSSE for the larger basis sets, since the differential BSSE effects on $T_{3}-\left(Q\right)$ and $\left(Q\right)$ pull in opposite directions.

The bottom line for thermochemical applications appears to be that BSSE contributions are negligible for even high‐accuracy work. Does this still bear out for polyatomics, or for noncovalent interactions?

#### 3.1.2 Small Polyatomics

While we would not be able to carry out cc‐pV5Z, let alone haV5Z CCSDT(*Q*) calculations on polyatomics, Table 3 presents results with smaller basis sets for a subset of about three dozen triatomics from the W4‐11 thermochemical benchmark [40]. Naturally, everything becomes larger in absolute numbers. Nevertheless, the same basic tendencies are seen as for the diatomics:

- BSSE on (*Q*) is basically insignificant and on ${\left(Q\right)}_{\Lambda }-\left(Q\right)$ wholly so.
- BSSE on $T_{3}-\left(T\right)$ skirts the 0.1 kcal/mol edge for cc‐pVDZ, but tapers down to 0.04 for cc‐pVTZ, and becomes negligible with the cc‐pV{D, T}Z extrapolation practiced in W4 theory and HEAT.

**TABLE 3:** RMS BSSE corrections (kcal/mol) to post‐CCSD(*T*) TAE_*e*_ contributions for a set of triatomics.

	(*T*)	${\left(T\right)}_{\Lambda }-\left(T\right)$	$T_{3}-\left(T\right)$	$T_{3}$	$\left(Q\right)$	*T*(*Q*) − (*T*)	${\left(Q\right)}_{\Lambda }-\left(Q\right)$
cc‐pVDZ	0.612	0.018	0.093	0.704	0.008	0.098	0.001
cc‐pVTZ	0.353	0.024	0.039	0.389	0.015	0.053	0.003
w/o ClOO	0.353	0.020	0.036	0.388	0.015	0.050	0.002
cc‐pV{D, T}Z^a^			0.017		0.019		
cc‐pVQZ	0.143	0.028	0.017	0.139	0.006		0.004
haVDZ	0.443	0.052	0.052	0.487	0.005		0.006
haVTZ	0.171	0.024	0.017	0.168	0.009		0.001

^a^ Extrapolation exponents from table 5 of Reference [47]. ClOO excluded.

We hence conclude that these thermochemical protocols require no modification to account for post‐CCSD(*T*) BSSE unless one targets an accuracy that is likely unattainable with W4‐ and HEAT‐type approaches.

And once again, substituting haV*n*Z for cc‐pV*n*Z cuts BSSE in half.

### 3.2 Noncovalent Interactions

#### 3.2.1 Small Noncovalent Dimers: The A24x8 Dataset

Counterpoise corrections data for the A24x8 dataset are summarized in Table 4.

**TABLE 4:** RMS BSSE corrections (kcal/mol) to post‐CCSD(*T*) $D_{e}$ contributions for the A24x8 set of noncovalent interactions.

	RMS ΔCP			RMS ΔCP/RMS $\Delta D_{e}$		
	(*T*)	$T_{3}-\left(T\right)$	$\left(Q\right)$	(*T*)	$T_{3}-\left(T\right)$	$\left(Q\right)$
cc‐pVDZ(no d)	0.060	0.003	0.004	0.890	0.205	0.239
cc‐pVDZ	0.067	0.002	0.003	0.759	0.162	0.208
cc‐pVTZ	0.049	0.001	0.003	0.290	0.082	0.134
cc‐pVQZ	0.027	0.002		0.127	0.080	
haVDZ	0.049	0.002		0.297	0.140	
haVTZ	0.016	0.003		0.074	0.147	
haVQZ	0.007			0.029		
haV5Z	0.003			0.014		

Noncovalent interactions are very different in their behavior from atomization energies, in that for most noncovalent complexes, in that MP2 is already a decent to good starting point (except for π‐stacking and related). Thus, CCSD‐MP2 and (*T*) are commonly evaluated using relatively small basis sets (see, e.g., References [41, 48] and references therein).

One might thus reasonably expect that post‐CCSD(*T*) contributions will be proportionally much smaller. Admittedly, of course, the A24x8 systems are quite small, and hence post‐CCSD(*T*) contributions might be somewhat less picayune in larger noncovalent complexes, especially at compressed geometries.

The RMS ΔBSSE values are even tinier in absolute terms: 0.002 kcal/mol for $T_{3}-\left(T\right)$ and 0.003–0.004 for (*Q*). For smaller basis sets, these are still nontrivial fractions of the actual $\Delta D_{e}$ contributions. Therefore, any post‐CCSD(*T*) corrections obtained with *very* small basis sets, such as the unpolarized double zeta cc‐pVDZ(no d), need to be regarded with some caution.

For a more reasonable cc‐pVTZ basis set, ΔBSSE represents about 8% of the ΔCP [$T_{3}-\left(T\right)$] and 13% of Δ(*Q*).

#### 3.2.2 Not‐So‐Small Noncovalent Complexes: The S66 Dataset

The aforementioned analysis is open to the criticism that the systems in A24 are quite small and not necessarily representative of what one might see in a real‐life application. In contrast, the well‐known S66 benchmark [41] consists of dimers of biomolecular building blocks interacting in different ways (hydrogen bonding, π‐stacking, pure London dispersion, and mixed‐influence). As such, it contains larger systems such as benzene dimer (both parallel‐displaced and T‐shaped, systems **24** and **47**, respectively), uracil dimer (both Watson‐Crick **17** and π‐stacked **26**), pentane and neopentane dimers (systems **34** and **36**, respectively).

For this dataset, we will alas have to limit ourselves to the cc‐pVDZ(no p on H), a.k.a., cc‐pVDZ(d, s), basis set. We were able to obtain full CCSDT BSSE corrections for 64 out of 66 systems, and CCSDT(*Q*) for about two dozen. (It bears reiterating that, while the dimers often posed memory or computation time requirements that exceeded our available resources, the evaluation of counterpoise corrections does *not* require the dimers *AB*, only the monomers in the full dimer basis set *A*(*B*) and *B*(*A*), as well as naturally the monomers in their own basis set.)

Some relevant statistics can be found in Table 5. Even though with this small basis set, the BSSE correction at the CCSD(*T*) level is quite hefty, the *differential* BSSE correction to $T_{3}-\left(T\right)$ is surprisingly modest, 0.018 kcal/mol RMS. In fact, the lion's share of even this small difference is recovered at the CCSDT‐3 level [49, 50, 51]. This approximate coupled cluster approach neglects the $T_{3}$ term in the $T_{3}$ amplitude equations, thus reducing computation time scaling with system size from the $O\left(n_{\mathrm{occ}.}^{3}N_{\text{virt}.}^{5}\right)$ of full CCSDT to the same $O\left(n_{\mathrm{occ}.}^{3}N_{\text{virt}.}^{4}\right)$ as CCSD(*T*). (The difference between CCSDT and CCSDT‐3 starts in fifth order in many‐body perturbation theory [52, 53], with the leading term $E_{\mathrm{TT}}^{\left[5\right]}$. The difference between CCSDT‐3 and CCSD(*T*), on the other hand, has the leading term $E_{\mathrm{TQ}}^{\left[5\right]}$ resulting from the action of the disconnected quadruples ${\hat{T}}_{2}^{2}/2$ on the connected triples amplitudes $T_{3}$.)

**TABLE 5:** RMS BSSE corrections (kcal/mol) to post‐CCSD(*T*) $D_{e}$ contributions for the S66 set of larger noncovalent complexes.

Difference	cc‐pVDZ(p, s)		cc‐pVDZ(d, s)	
	RMSDiff(A, B)	$N_{s\text{ystems}}$	RMSDiff(A, B)	$N_{s\text{ystems}}$
CCSD–nihil	3.382	66	3.290	66
CCSD(*T*)–CCSD	0.211	66	0.232	66
CCSDT‐3–CCSD(*T*)	0.013	66	0.018	66
CCSD(*T*)_Λ_–CCSD(*T*)	0.009	66	0.010	66
CCSDT–CCSD(*T*)	0.008	66	0.013	64^a^
CCSDT–CCSD(*T*)_Λ_	0.007	66	0.006	64
CCSDT–CCSDT‐3	0.007	66	0.007	64
CCSDT(*Q*)–CCSDT	0.007	59	0.008	25
CCSDT(*Q*)–CCSDT‐3	0.011	59	0.014	25
CCSDT(*Q*)–CCSD(*T*)	0.003	59	0.005	25

^a^ Missing S66 systems **41** uracil‐pentane and **43** uracil‐neopentane.

A still more economical approximation is offered by CCSD(*T*)_Λ_ [34, 35, 36, 37] which is only $O\left(n_{\mathrm{occ}.}^{2}N_{\text{virt}.}^{4}\right)$ in the iterations, followed by a single $O\left(n_{\mathrm{occ}.}^{3}N_{\text{virt}.}^{4}\right)$ step. Its cost premium over CCSD(*T*) is just in the need to also solve for the “left‐hand eigenvectors” aside from the CCSD “right‐hand” solution (which approximately doubles overall CPU time).

While we admittedly do not have as many data points for (*Q*) as for $T_{3}-\left(T\right)$, for the available ones the ΔCP is just 0.008 kcal/mol RMS. What is more, $\mathrm{\Delta CP}\left[T_{3}-\left(T\right)\right]$ and $\mathrm{\Delta CP}\left[\left(Q\right)\right]$ have opposite signs (like the underlying contributions) and cancel each other to a large degree. As a result, the cumulative $\mathrm{\Delta CP}\left[\text{CCSDT}\left(Q\right)-\text{CCSD}\left(T\right)\right]$ is just a measly 0.006 kcal/mol, which can be regarded as negligible by any reasonable standard.

If we remove all polarization functions from cc‐pVDZ, we are left with just a split‐valence basis set, and most of the CCSDT(*Q*) calculations come within reach. To our astonishment, we found that the differential BSSEs on $T_{3}-\left(T\right)$, (*Q*), and CCSDT(*Q*)–CCSD(*T*) remain equally tiny.

## 4 Conclusions

In response to our research question, we can conclude the following:

1. For high‐accuracy computational thermochemistry, particularly total atomization energies obtained at the W4 or HEAT levels, $T_{3}-\left(T\right)$ with the cc‐pVDZ basis set carries a small but noticeable BSSE.
2. This is effectively removed, however, by the extrapolation of $T_{3}-\left(T\right)$ from cc‐pV{D, T}Z basis sets.
3. BSSE on (*Q*) may be regarded as negligible in a thermochemistry context.
4. For noncovalent interactions, BSSE on both $T_{3}-\left(T\right)$ and $\left(Q\right)$ is insignificant even for basis sets as small as cc‐pVDZ, and besides is subject to a degree of mutual cancelation between $T_{3}-\left(T\right)$ and (*Q*).

## Author Contributions

M.S. brought on the subject matter. V.F. performed the calculations and curated the data for the TAE BSSE corrections. E.S. carried out the A24x8 calculations and curated the relevant data. E.S. and J.M.L.M. shared the S66 calculations. J.M.L.M. oversaw the project and wrote the first draft, while all authors contributed to the completed manuscript.

## Conflicts of Interest

The authors declare no conflicts of interest.

## Supporting information


Data S1.

Table S1.

Table S2.

Table S3.

Table S4.

Table S5.

## Data Availability

The data that support the findings of this study are available in the Supporting Information of this article.
